# The temporal dynamics of resting-state EEG microstates reflected the differences in socioeconomic status among college students

**DOI:** 10.7717/peerj.20697

**Published:** 2026-01-30

**Authors:** Qidan Ren, Fangfang Long, Yunlu Xie, Huiling Chen, Ying Jiang

**Affiliations:** School of Psychology, Guizhou Normal University, Guiyang, Guizhou, China

**Keywords:** EEG microstate, Socioeconomic status, Resting-state, Large-scale neural network, Brain dynamics

## Abstract

**Background:**

Socioeconomic status (SES) is a distal ecological factor that predicts the trajectory of human development. Exposure to low SES may have lasting effects on brain structure and function. Although prior research has identified static neural correlates of SES disparities, it remains unclear how socioeconomic contexts shape dynamic brain states. Therefore, the present study employs electroencephalography (EEG) microstate analysis to investigate how SES influences the dynamics of resting-state brain activity.

**Methods:**

Based on SES scores, participants in the top and bottom 27% were categorized as the high-SES group (*n* = 29), and the low-SES group (*n* = 29). Resting-state EEG signals were collected from all participants, and microstate analysis identified the temporal features of four canonical large-scale neural networks (microstates A, B, C, and D) to explore socioeconomic differences in brain dynamics across different SES groups.

**Results:**

(1) The correlation between SES and the temporal characteristics of both microstates A (*ps* < 0.05) and C (*ps* < 0.05) was significant, suggesting that SES may be associated with neural dynamics involved in auditory-language processing and the default mode network (DMN). (2) High- and low-SES groups exhibited divergent temporal characteristics in microstate dynamics. Compared with the high-SES group, participants in the low-SES group demonstrated larger duration (*p* = 0.025), occurrence (*p* = 0.002), and time coverage (*p* < 0.001) in microstate A, while exhibiting reduced occurrence (*p* < 0.001) and time coverage (*p* = 0.005) in microstate C. The results indicate that the low-SES individuals may have compensatory reinforcement of the auditory-language network and a weakened DMN activity. (3) High- and low-SES groups exhibiting different microstate transition patterns may reflect distinct cognitive control mechanisms. Compared with the high-SES group, the low-SES group demonstrated that the transition probabilities between microstates A and B (*ps* < 0.05), A and D (*ps* < 0.05) were significantly higher, whereas those between microstates B and C (*ps* < 0.05), C and D (*ps* < 0.05) were significantly lower.

**Conclusion:**

These findings reveal a robust association between SES disparities and spatiotemporal EEG microstate dynamics. The reconfiguration of metastable brain states may represent the way the brain responds to challenging environments.

## Introduction

Socioeconomic status (SES) is a multidimensional construct reflecting an individual’s access to material and social resources as well as their position in societal hierarchies, and serves as a critical environmental predictor of language and cognitive development (Thomas, Forrester & Ronald, 2013). Convergent evidence indicates that socioeconomic disparities modulate neural circuit refinement, particularly during sensitive developmental windows (Malave, van Dijk & Anacker, 2022), with enduring consequences for cognitive functioning and behavioral problems (Wang et al., 2023). Notably, studies reveal SES-related neural processing differences even in the absence of behavioral performance gaps, suggesting compensatory mechanisms at the neurobiological level (Hackman & Farah, 2009; Ursache & Noble, 2016). Such neuroplastic adaptation occurs when individuals are frequently exposed to a challenging environment (Hackman, Farah & Meaney, 2010), characterized by structural and functional reorganization in response to environmental demands (Noble et al., 2015). However, previous studies predominantly focus on static neural correlates of SES disparities, it remains unclear how socioeconomic contexts shape dynamic brain states. Therefore, the present study aims to investigate how individuals from different SES backgrounds exhibit distinct patterns of neural dynamics as they respond to their specific environmental demands, providing novel insights into the spatiotemporal dynamics underlying SES-related neural alterations.

Recent advances in neuroimaging methods have made accessible new ways of disentangling the impacts of SES on brain development. Previous research has revealed SES-related disparities in language and cognitive abilities among individuals, which emerge early and demonstrate persistence. For example, SES-related disparities in language processing and cognition may already be present in infancy and are still observed in young adulthood (Brown, 2021; Fernald, Marchman & Weisleder, 2013; Lyu & Burr, 2016). Neuroimaging evidence reveals that these behavioral outcomes are the result of structural and functional brain adaptations shaped by socioeconomic contexts. Specifically, individuals from low SES backgrounds often processed incoming speech less efficiently and took longer to identify the meaning of a word in the context of sentences and conversations (Fernald, Marchman & Weisleder, 2013). Researchers found the explanation that individuals from low-SES backgrounds had reduced temporal lobe volume and smaller cortical surface area in the temporal region (Loued-Khenissi et al., 2022), indicating weaker hemispheric specialization for language processing and less efficient functional network organization (Chan et al., 2018; Raizada et al., 2008). In the domain of executive function, reduced cognitive control and greater impulsivity in low-SES individuals have been related to diminished structural integrity and activation of the prefrontal cortex (Colich et al., 2020; Murtha et al., 2023). While these studies underscore the impact of SES on discrete brain regions, such region-specific findings may offer only a partial account of SES-related neural differences. Given that SES fundamentally constitutes a multidimensional social construct rather than a neuropathological entity analogous to focal brain lesions. The nature of SES implies the relationship between SES and functional brain activity is not specific to a few neurocognitive systems or individual brain regions (Perera et al., 2021). The SES effect is more plausibly reflected in the distributed large-scale neural networks (Sripada et al., 2014). Therefore, to understand how the brain implements adaptive processes in response to environmental demands, adopting a network-level perspective could provide valuable insights.

Empirical support for this idea comes from studies examining SES-related differences in large-scale neural networks. For instance, SES disparities in language exposure have been linked to altered functional connectivity in the auditory-language network (Biazoli et al., 2020; Romeo et al., 2018). Additionally, the default mode network (DMN) has been shown to mediate SES-related differences in cognitive control and self-referential processing (Assari et al., 2025; Tomiyama et al., 2022). One proposed explanation points to within-network connectivity differences in the DMN among individuals of different SES (Olson, Chen & Fishman, 2021). However, these findings are primarily based on functional magnetic resonance imaging (fMRI) or structural magnetic resonance imaging (MRI) studies, which offer limited insight into the temporal fluctuations of brain networks. As a result, it remains unclear how these networks activate, deactivate, and transition across time in the context of SES. To quantify the impact of SES on brain functional dynamics, the present study utilizes analytical approaches that can resolve the dynamic spatiotemporal characteristics of neural networks.

To address this methodological gap, electroencephalography (EEG) microstate analysis has been proposed as a promising approach to investigate the real-time dynamics of large-scale neural networks during development. EEG microstate analysis is one of the whole-brain imaging approaches that could characterize the spatial organization and temporal dynamics of large-scale neural networks, offering new opportunities for investigating the impact of SES on moment-to-moment brain states. In previous studies, the four microstate categories have been widely adopted because of consistent and reproducible map topographies, the capacity to reflect core large-scale neural networks, and consistent dominance. First, EEG microstates are consistently categorized into four stable, canonical classes. The four classical types named microstate A, B, C, and D, which could account for 80% of the total EEG data variance (Van de Ville, Britz & Michel, 2010). The four categories of topographies are stable and reproducible across different populations and the entire life span (Britz, Van DeVille & Michel, 2010). Second, each microstate is thought to represent the transient activation of a specific large-scale neural network (Britz, Van DeVille & Michel, 2010; Yuan et al., 2012). Specifically, microstate A is associated with the auditory-language network involving semantic or phonological processing, microstate B is related to visual and imagery processing, microstate C is related to DMN, and microstate D is related to the dorsal attentional network (Britz, Van DeVille & Michel, 2010). Third, even if more cluster microstates are selected, these four microstates seem to consistently dominate. This dominance is supported by research proposing that the brain networks of other microstates likely overlap with sources for the four fundamental classes (Custo et al., 2017; Tarailis et al., 2024). Based on these three reasons, the present study focused on four classes of microstates that could provide a concise and comprehensive approach to explore the SES effects on brain dynamics.

Recent advancements in microstate research have extended beyond classifying canonical map types, emphasizing instead the temporal parameters and sequential transitions between microstates. These dynamic features, including duration, occurrence, time coverage, and transition probabilities, offer richer insight into the real-time coordination of large-scale neural networks and their links to diverse cognitive processes and psychological states (Rieger et al., 2016; Seitzman et al., 2017). For example, increased duration and coverage of microstate A have been observed during tasks requiring sustained sound localization and continuous phonological processing (Cui et al., 2021; Tomescu et al., 2022). Similarly, a decrease in the occurrence or coverage of microstate C has been considered an indicator of impaired activity in functional networks. On the one hand, the reduced occurrence of microstate C in individuals with cognitive decline was interpreted as a decrease in connectivity of the DMN (Jabes et al., 2021). On the other hand, a reduction in the occurrence of microstate C was observed in individuals with low subjective socioeconomic status (SSS) has been interpreted as a decrease in emotional control function (Guo et al., 2020). Moreover, transitions between microstates follow non-random patterns that differ across individuals and psychological traits. For instance, individuals with low SSS and high neuroticism showed a stronger transition probability from microstate A to C, suggesting that SES-linked psychological characteristics may be encoded in microstate switching patterns (Guo et al., 2020). These findings of microstate transition characteristics support the idea that sequential microstate dynamics reflect individual-level differences in brain network coordination and environmental adaptation.

While previous studies have offered valuable insights, their predominant focus on SSS has left SES largely unexplored in microstate research. SSS and SES are distinct in both the definitions and assessments. Specifically, SSS reflects an individual’s perceived social position and emotion-related subjective evaluation. In contrast, SES is typically measured using objective indicators such as income, educational attainment, and occupational status (Lyu, Peng & Hu, 2019). Additionally, SSS involves personal evaluations and is closely linked to emotion and psychological experiences (Ma et al., 2024). However, SES primarily influences brain development through exposure to adversities such as material deprivation and environmental unpredictability, which are believed to have a stronger association with brain development (Noble et al., 2015). SES holds a distinct advantage in investigating brain development due to its direct links to concrete adversities. While previous research has examined the psychological pathways linking SSS to brain network dynamics (Guo et al., 2020), the specific environmental mechanisms through which SES exerts its influence remain unclear. Therefore, the current study investigates EEG microstate dynamics in relation to SES, providing a novel perspective for understanding how socioeconomic conditions influence real-time brain activity at the level of large-scale neural network coordination. Building on this foundation, the present study aims to examine how SES influences the spatiotemporal dynamics of resting-state brain activity, as indexed by EEG microstate parameters. By focusing on both microstate-specific temporal features and transition probabilities, we seek to identify distinct neurodynamic patterns that may underlie SES-related adaptations in brain function.

In general, EEG microstate analysis provides a promising approach to explore the potential pathways through which SES may shape brain dynamics. Based on previous findings and theoretical models of neuroplasticity and environmental adaptation, we propose the following hypotheses: (1) low SES individuals are less efficient in language processing, which would be reflected in larger duration and time coverage of microstate A related to speech processing; (2) if microstate C reflects transient engagement of the DMN, as previous research suggests, then a reduced occurrence of microstate C in low SES individuals may indicate attenuated DMN involvement during rest; and (3) SES-related differences in environmental adaptation may be indexed by distinctive patterns of network sequential activation, as reflected in transition probabilities between microstates, particularly in the dynamic interplay between microstates A and C.

## Materials & Methods

### Participants

Based on calculations using G*Power 3.1 and referencing the effect sizes from a similar previous study in the microstate domain (Bai, Yu & Li, 2024), an *F* test with a medium effect size *f* = 0.25 and a significance level of 0.05 was required to achieve a statistical power of 0.80 with a total sample size of 24 participants, with 12 participants in each group. As a multidimensional construct, SES lacks standard thresholds for categorization. Thus, the extreme groups approach was chosen to categorize individuals into high and low SES levels. This approach has been frequently employed in previous studies (Ye, Huang & Liu, 2021). Considering the possible sample loss, an initial 108 participants participated in this study through recruitment posters. One participant was excluded because of poor EEG data quality. According to the top and bottom 27% of their SES scores, the participants were divided into two groups as high-SES (18 females, average age 19.41 ± 1.72 years) and low-SES (19 females, average age 19.86 ± 1.60 years), with 29 subjects in each group. All participants were right-handed, had normal or corrected-to-normal vision, and had no history of neurological or psychological impairment. The study protocol was approved by the Human Subjects Protection Committee at Guizhou Normal University, School of Psychology (Approval number: GZNUPSY.N.202411E [0040]). All participants signed informed consent and received monetary compensation upon completion.

## Materials

### Socioeconomic status

Considering college students are not familiar with their exact household income (Shi & Shen, 2007), SES was measured on the basis of the education and the occupation reported by the participants for their parents (De Cat, 2021; Shi & Shen, 2007). Parental education was assessed using a 5-point scale ranging from 1 = primary school education or less to 5 = graduate degree. Parental occupation was measured on a 10-point scale ranging from 1 = unemployment to 10 = state and social managers (Zhou & Guo, 2013). The SES score of each participant was calculated by first averaging the education and occupation scores of both parents to one family education score and one family occupation score, respectively. The two scores were then transformed into Z-scores and summed to produce the final SES measure (Wang & Liu, 2021). The low-SES (*M* =  − 1.61, *SD* = 0.36) and high-SES (*M* = 2.28, *SD* = 1.58) groups showed a significant difference in their SES scores (*t* =  − 12.89, *p* < 0.001).

### EEG recording and preprocessing

The EEG data are collected in the afternoon. To ensure good data quality, the participants took a 10-minute break before starting the EEG data collection after arriving at the laboratory. During the EEG recording, the participants were asked to view a fixation cross in the center of the computer screen and were instructed to alternately open and close their eyes. About 8 min of EEG data were recorded in a sound- and electrically-shielded room using a 64 Ag/AgCl scalp site according to the international 10–20 system in an elastic cap (Neuro Scan Product). Throughout this time, they were asked to relax, remain awake, and avoid engaging in any specific thoughts. The left mastoid process was used as the reference electrode during online recording, and the ground point was located at the midpoint of the connection between the FPz and Fz electrodes in the front middle of the scalp. The sampling frequency was 1,000 Hz, the filter bandpass was 0.05∼1,000 Hz, and the scalp impedance was less than 10 kΩ.

EEG data with eyes closed were used for subsequent analyses, as eyes-closed EEG data are known to provide more reliable measures of resting-state brain activity (Barry et al., 2007). EEG data were pre-processed using EEGLAB, an open-source toolbox running in the MATLAB environment, and in-house MATLAB functions. Initial steps included channel positioning, deletion of non-useful electrodes, bandpass filtering (1∼80 Hz), notch filtering (48∼52 Hz), conversion of unilateral mastoid reference to bilateral mastoid reference, and segmenting into 2-s epochs. Subsequent steps involved manual data inspection for poor electrodes and their interpolation using the spherical spline method, removing segments with severe drift, applying Independent Component Analysis (ICA) to remove artifacts like sweat and eye movements, and removing extreme values (amplitudes exceeding ±80 µV).

### EEG microstate analysis

Before microstate analysis, the remaining EEG epochs were digitally band-pass filtered between 2 and 20 Hz as suggested by previous studies (Schlegel et al., 2012), and were re-referenced to the whole-brain average.

Microstate analyses consist of the following steps. First, the Global Field Power (GFP), defined as the EEG potential variance across scalp electrodes, was computed for each time point (Lehmann & Skrandies, 1984). Since the EEG topographies remain stable around peaks of GFP and change during the troughs, only the topographies at peaks of GFP were further analyzed. Then, to determine the optimal number of categories for the two SES groups and the corresponding template for each category, the Atomize-Agglomerate Hierarchical cluster (AAHC) algorithm was used to analyze the data of each participant in the low SES group and the high SES group separately, without considering the polarity of each topographic map. Second, a fixed number of four clusters was chosen. These group average template maps were sorted according to the standard labelling (microstate A: left–right orientation; microstate B: right-left orientation; microstate C: anterior-posterior orientation; microstate D: fronto-central maximum) (Tarailis et al., 2024). Third, to obtain each individual’s microstate characteristics for statistical analyses, previously identified across different SES groups’ individual maps were sorted. This was done separately for both groups (*i.e.,* low SES and high SES) on the basis of spatial correlations using the templates. Finally, the GFP peaks of the individual EEG data were assigned to the best-fitting individually identified cluster maps. This assignment was linearly interpolated to the time period between the GFP peaks in order to obtain a continuous temporal stream of microstates occurring in each individual.

From this last step, four microstate features were extracted for each participant in the high and low SES conditions: (1) the average duration of each microstate (duration); (2) the frequency of occurrence (occurrence, the average number of occurrences of each microstate category per second); (3) time coverage (the total duration of each microstate category as a percentage of the total resting EEG); (4) the transition probability (the probability of transition from one microstate category to another).

### Statistics analysis

All statistical analyses were performed using IBM SPSS software. Pearson correlation analyses were conducted to examine the relationship between SES scores and microstate parameters (duration, occurrence, and time coverage). Separate two-way analyses of covariance (ANCOVAs) were performed for the three microstate parameters (duration, occurrence, and time coverage). Each ANCOVA contained one between-subject factor (groups: high and low SES groups), one within-subject factor (microstate categories: microstate A, B, C, and D), and two covariates for age and gender of two groups, since previous studies suggested that these parameters may be modulated by age (Koenig et al., 2002; Niu et al., 2024). When the main effects or interaction were significant, followed by *post hoc t*-tests with Bonferroni correction for age and gender as covariates were conducted. Independent samples *t*-tests were used to analyze the transition probabilities.

## Results

### Microstate topographies

**Figure 1:**
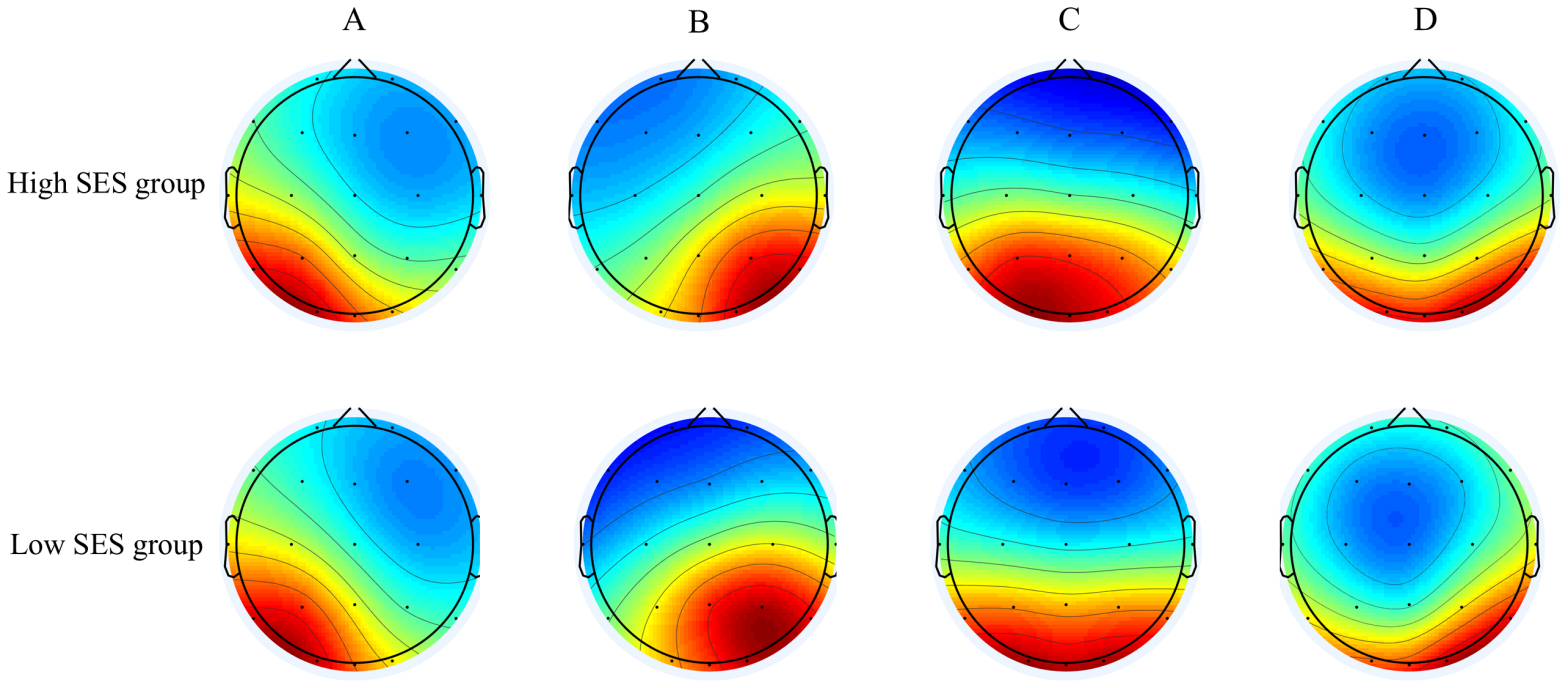
Differences in the group average template maps of the four microstate categories between the high and low SES groups.

To maximize comparability with previous research, applying the AAHC clustering algorithm, four archetypal microstate topographies A-D for the high and low SES groups were identified separately (see Fig. 1). The Global Explained Variance (GEV) is a metric that quantifies the extent to which microstates account for the variance in EEG data. In this study, the GEV of the four microstates was 79.44% (*SD* = 4.36%) in the high SES group and 78.31% (*SD* = 3.25%) in the low SES group, showing robust representativeness across both socioeconomic groups.

### Correlation between SES and microstate parameters

**Figure 2:**
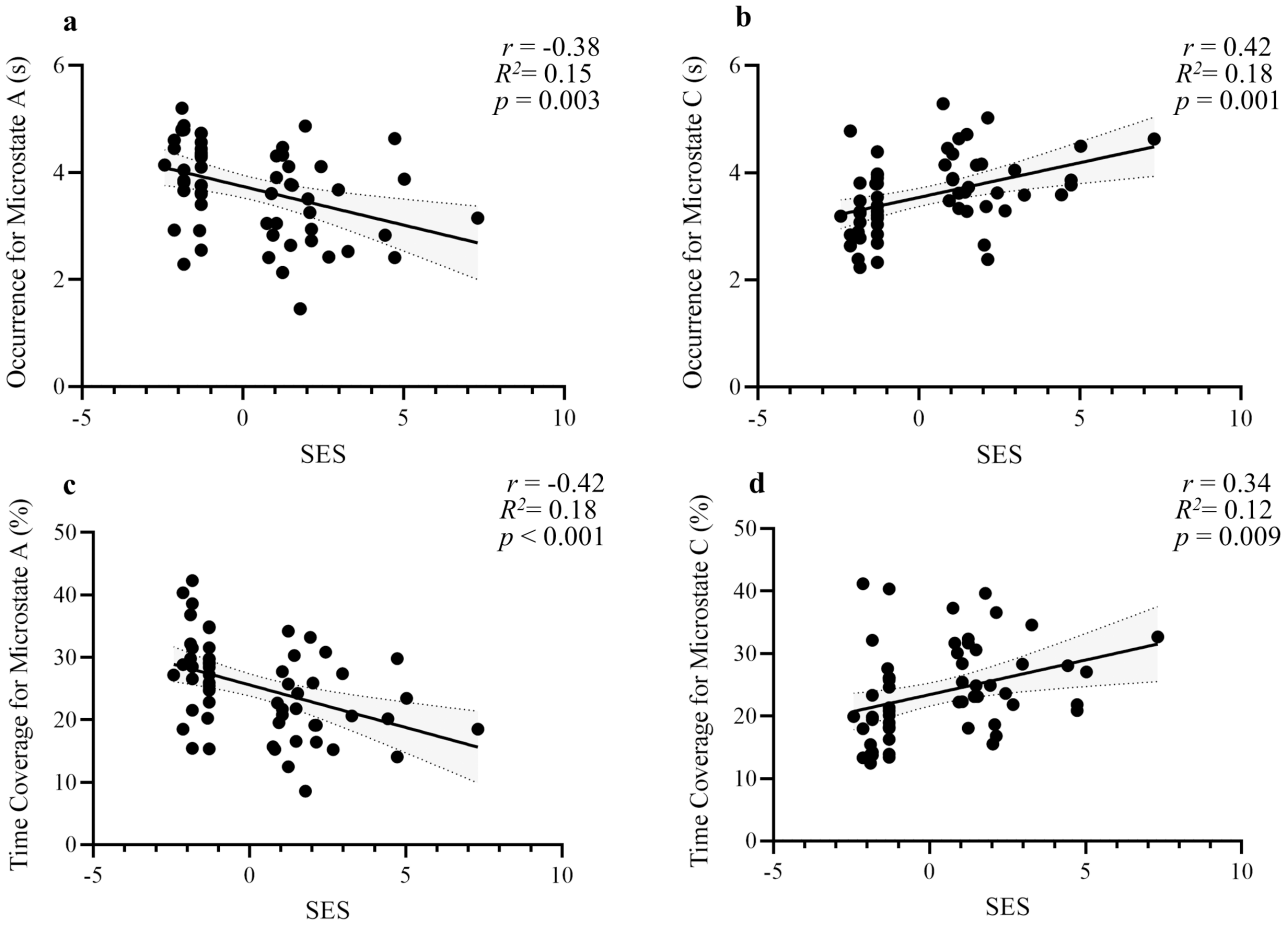
Relationship between SES and microstate parameters. Scatterplots showing associations between SES score and (A) occurrence of microstate A, (B) occurrence of microstate C, (C) time coverage of microstate A (%), and (D) time coverage of microstate C (%), all with 95% confidence intervals.

Correlation analyses were conducted to examine the relationship between SES and three microstate parameters. The results are shown in Fig. 2. For microstate duration, no significant correlation was found between SES and the duration of the four microstates. For microstate occurrence, SES was negatively correlated with the occurrence of microstate A (*r* =  − 0.38, *p* = 0.003) and positively correlated with the occurrence of microstate C (*r* = 0.42, *p* = 0.001). For microstate time coverage, SES was negatively correlated with the time coverage of microstate A (*r* =  − 0.42, *p* < 0.001), and SES was positively correlated with the time coverage of microstate C (*r* = 0.34, *p* = 0.009). No significant correlation was found between SES and the parameters of microstate B (*r* = 0.01∼0.15, *p*s > 0.05) or D (*r* = 0.04∼0.15, *p*s > 0.05). Taken together, the results showed that SES was closely related to activation of the auditory-language network and neural activity in speech or semantic processing, and was also associated with increased frequency of activation in the DMN and enhanced self-image at the neural level.

### The differences in microstate parameters between high- and low-SES groups

**Figure 3:**
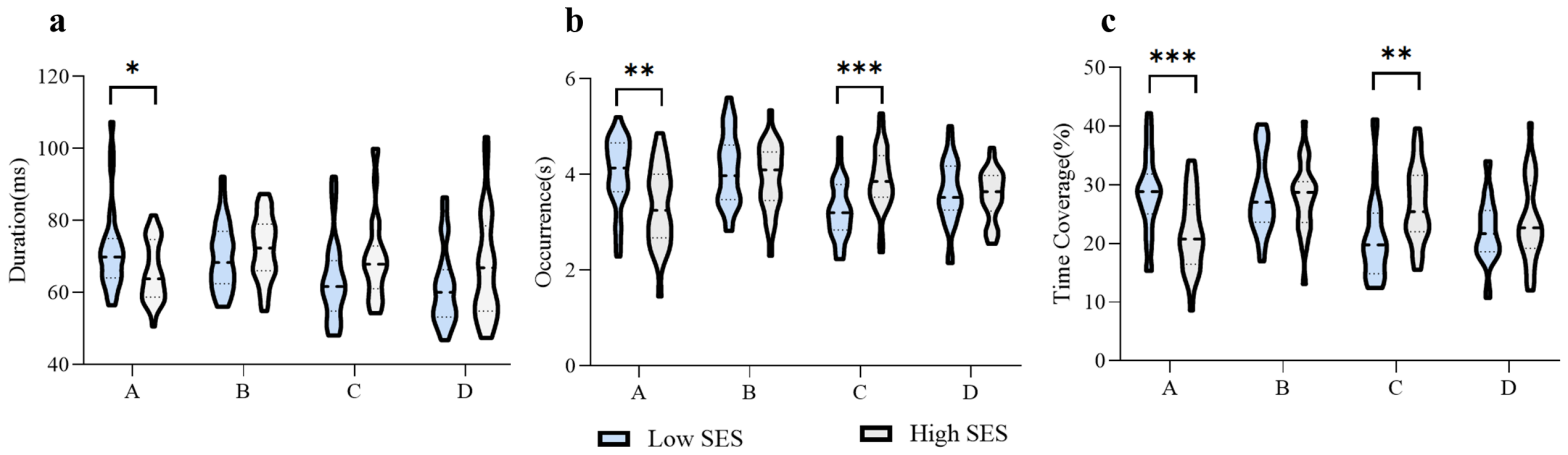
Characteristics of temporal parameters of EEG microstates between different SES groups. The shape of the violin plot indicates the density of the data distribution. The three black lines in the violin plot denote the upper quartile, the median, and the lower quartile. ***p < 0.001; **p < 0.01; *p < 0.05.

In order to reveal the characteristic patterns of the differences between high and low SES groups on specific microstate parameters (duration, occurrence, and time coverage), separate two-way analyses of covariance (ANCOVAs) were conducted. For microstate parameter duration of a microstate, the results found that (1) the main effect of the microstate category was not significant, *F*(3, 162) = 0.62, *p* = 0.604; (2) the main effect of group was not significant, *F*(1, 54) = 0.68, *p* = 0.414; (3) while the interaction between group and microstate category was significant, *F*(3, 162) = 4.17, *p* = 0.007, ${\eta }_{\mathrm{p}}^{2}=0.07$. *Post hoc* tests found that the duration of microstate A was longer in the low SES group compared to the high SES group after Bonferroni correction (*p* = 0.025) (Fig. 3A), indicating the stability of microstate A in the group with a low SES background.

For microstate parameter occurrence of a microstate, the result found that (1) the main effect of the microstate category was not significant, *F*(3, 162) = 1.72, *p* = 0.165, (2) the main effect of group was not significant, *F*(1, 54) = 0.36, *p* = 0.554, (3) the interaction between group and microstate category was significant, *F*(3, 162) = 8.45, *p* < 0.001, ${\eta }_{\mathrm{p}}^{2}=0.14$. *Post hoc* tests revealed that microstate A occurred more frequently (*p* = 0.002) and microstate C less frequently (*p* < 0.001) in the low SES group than in the high SES group (Fig. 3B), suggesting larger level of activation of the auditory-language network and the opposite in the DMN in the low SES group.

For microstate parameter time coverage of a microstate, the results found that (1) the main effect of the microstate category was not significant, *F*(3, 162) = 1.42, *p* = 0.240, (2) the main effect of group was not significant, *F*(1, 54) = 2.23, *p* = 0.141, (3) the interaction between group and microstate category was significant, *F*(3, 162) = 6.10, *p* < 0.001, ${\eta }_{\mathrm{p}}^{2}=0.10$. *Post hoc* tests revealed that compared with the high SES group, the low SES group had larger time coverage of microstate A (*p* < 0.001) and lower coverage of microstate C (*p* = 0.005) (Fig. 3C), suggesting neural activity in the auditory-language network is more sustained and the opposite in the DMN in the low SES group.

### Probability of transition between four microstates

**Figure 4:**
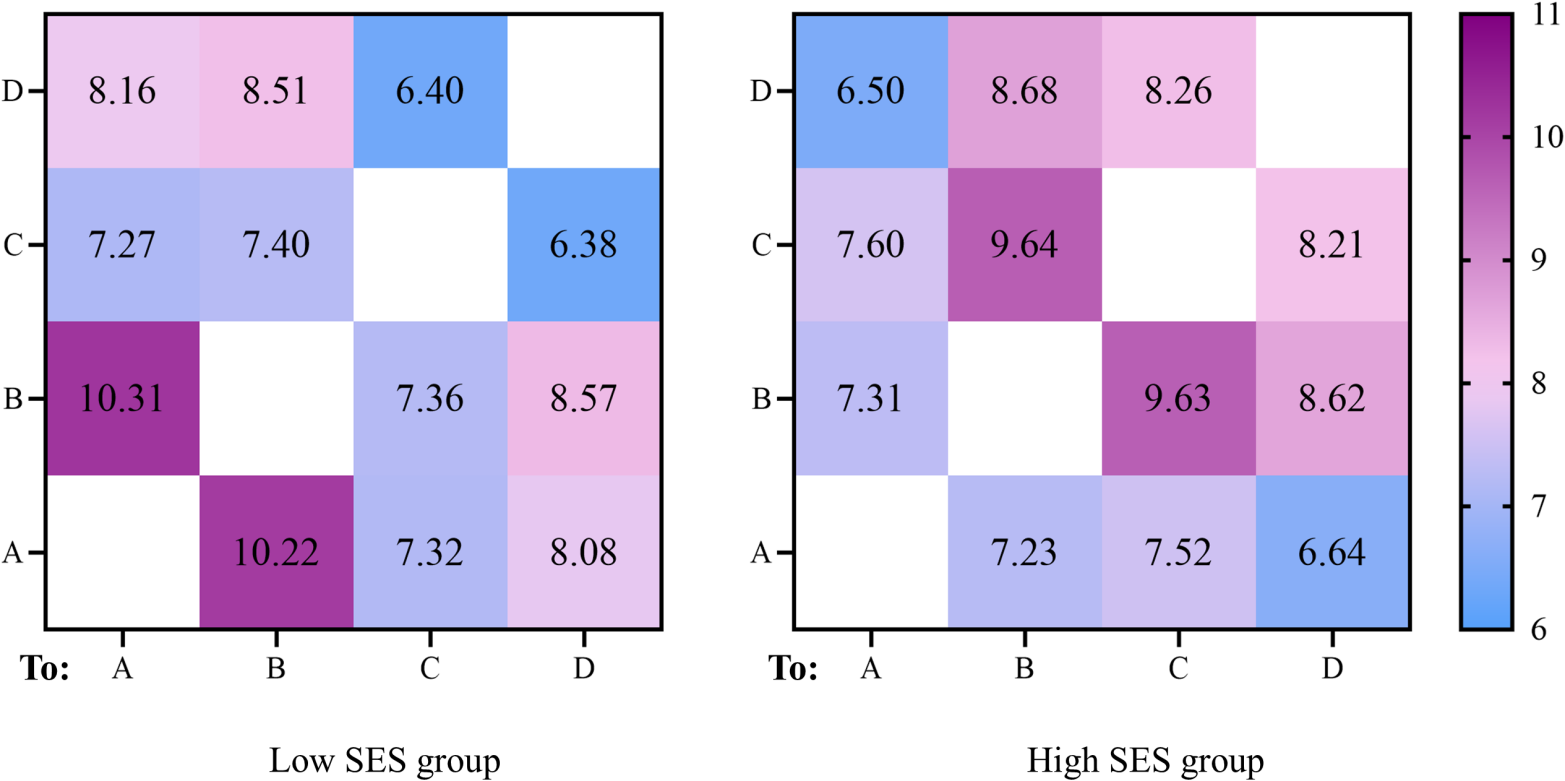
Probability of transition between microstates of different SES groups (%).

**Figure 5:**
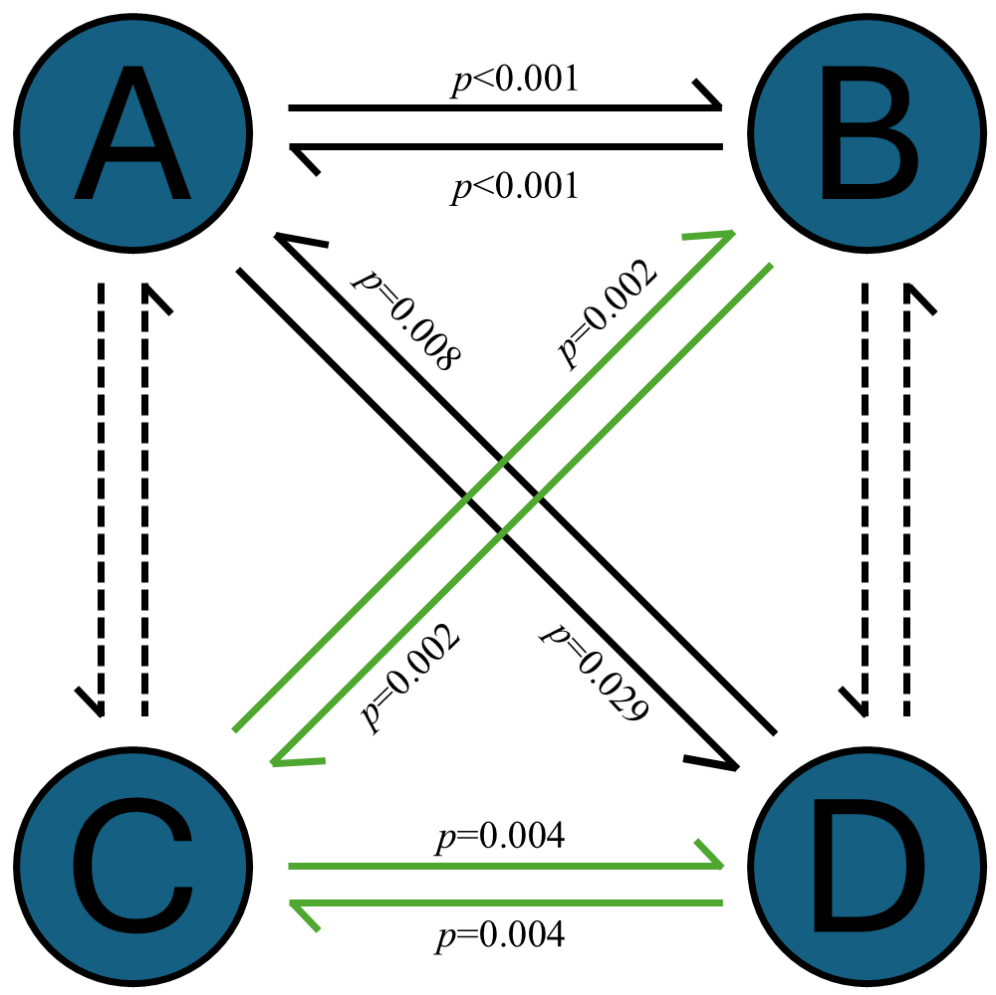
Differences in microstate transition probabilities between different SES groups. Solid lines represent *p* < 0.05, dashed lines represent *p* > 0.05; the black solid line indicates that the low SES group had significantly higher microstate transition probability than the high SES group. While the green solid line indicates that the high SES group had significantly higher microstate transition probability than the low SES group.

In order to explore the distinctive patterns of networks sequential activation caused by differences in environmental adaptation, the mean value of the transition probabilities for each microstate was calculated separately for the high and low SES groups, as shown in Fig. 4. An independent sample *t*-test was used to compare the transition probabilities of microstates between high and low SES groups. The results found that the transitions from microstate A to B (*t* = 4.28, *p* < 0.001) and B to A (*t* = 4.30, *p* < 0.001) were significantly higher in the low SES group than in the high group, and transitions from microstate A to D (*t* = 2.24, *p* = 0.029) and D to A (*t* = 2.73, *p* = 0.008) were significantly higher in the low SES group than in the high SES group. In contrast, transitions from microstate B to C (*t* =  − 3.29, *p* = 0.002) and C to B (*t* =  − 3.21, *p* = 0.002) were significantly lower in the low SES group than in the high SES group, and transitions from microstate C to D (*t* =  − 2.99, *p* = 0.004) and D to C (*t* =  − 3.03, *p* = 0.004) were significantly lower in the low SES group than in the high SES group (Fig. 5). In general, the results of transition probabilities suggest that living conditions shape the directional predominance of transitions between microstate categories.

## Discussion

The present study used EEG microstate analysis to investigate the differences in temporal dynamics of large-scale neural networks during resting-state between individuals from low SES and high SES backgrounds. The findings showed that the high and low SES groups differ in temporal dynamics between microstates A and C. Compared with the high-SES group, the low-SES group showed larger duration, occurrence, and time coverage in microstate A, while exhibiting reduced occurrence and time coverage in microstate C. Furthermore, distinct sequential network activation patterns were observed across different SES groups. This exploratory study provides initial evidence supporting the utility of resting-state EEG microstate metrics as potential neurophysiological markers of socioeconomic influences on brain development.

The results showed that the duration, occurrence, and time coverage of microstate A were significantly larger in the low SES group than in the high SES group, partially supporting hypothesis 1. Due to microstate A reflecting the auditory-language network (Britz, Van DeVille & Michel, 2010), it is possible that the observed result suggests a more stable, frequently activated, and persistently engaged auditory-language network in individuals from lower-SES backgrounds (Zhu & Gong, 2023). One possible interpretation of this pattern is that it might represent a broader neurocognitive mechanism characterized by compensatory recruitment in response to insufficient language input in low-SES individuals (Hackman & Farah, 2009). Specifically, the increased duration of microstate A may reflect less efficient information processing, marked by prolonged engagement of language-related circuits (Cui et al., 2021; Tomescu et al., 2022). The higher occurrence suggests frequent reactivation of the auditory-language network, possibly due to difficulties in maintaining sustained linguistic representations (Zhu & Gong, 2023). The increased time coverage of microstate A indicates extended neural resource allocation to speech-related processing, pointing to reduced neural efficiency (Cui et al., 2021). This difference was not observed between high and low SSS groups (Guo et al., 2020). The distinct effect of SES may stem from its demonstrated stronger association with brain function through the environment to which it is exposed (Noble et al., 2015). In contrast, SSS is more influenced by psychological factors and is likely connected to emotional networks rather than auditory. Together, these findings may indicate that reduced quality and quantity of language exposure among low-SES individuals leads to the development of a compensatory strategy to alleviate the difficulties in semantic processing (Fernald, Marchman & Weisleder, 2013; Raizada et al., 2008; Kornilov et al., 2019). Such compensatory patterns may reflect an adaptive mechanism whereby low-SES individuals attempt to stabilize language comprehension through greater and longer-lasting recruitment of auditory-language networks. Nevertheless, in the lack of phonological processing ability assessments, the compensatory strategies may be considered speculative and need validation from future research to explore.

As for microstate C, the parameters showed different patterns. Only the occurrence and time coverage of microstate C were significantly lower in the low SES group than in the high SES group, which was consistent with hypothesis 2. As microstate C was associated with the DMN (Britz, Van DeVille & Michel, 2010), the findings indicated a possible tendency for DMN underactivation and diminished engagement in individuals from lower-SES backgrounds. The decrease of microstate C may be linked to a weakening or suppression of DMN function in low-SES individuals. The DMN plays a crucial role in various internally-focused cognitive processes, including self-referential thinking and the construction of mental simulations (Tomescu et al., 2022; Zanesco, Denkova & Jha, 2021). Consequently, reduced occurrence and time coverage of microstate C may indicate limitations in self-relevant or introspective processing among low-SES individuals. One possibility is that individuals with low SES facing financial hardship may prioritize allocating cognitive resources towards immediate survival needs. This prioritization of cognitive toward urgent concerns might occur at the expense of internal processing necessary for long-term planning (Shah, Mullainathan & Shafir, 2012). Specifically, the decreased occurrence of microstate C indicates fewer activations of DMN, possibly due to reduced DMN connectivity caused by socioeconomic disadvantage environments unfavorable for synapse formation (Olson, Chen & Fishman, 2021; Abo Hamza et al., 2024). The lower time coverage signifies a smaller proportion of resting-state brain activity devoted to these introspective cognitive processes in individuals with low SES. The results align with previous studies reporting weakened DMN connectivity and reduced activation levels in low-SES individuals (Jabes et al., 2021; Assari et al., 2025). Furthermore, the result extended previous research, which observed a similar reduction in those with low SSS (Guo et al., 2020). SES and SSS cause similar alterations in brain networks may stem from both being linked to social position and shared neural mechanisms (Ursache & Noble, 2016). Microstate C is related to the cingulate cortex and prefrontal cortex, which are involved in emotional regulation and cognition (Britz, Van DeVille & Michel, 2010). Socioeconomic disadvantage may weaken the function of this network through mechanisms like stress and resource scarcity, leading to a reduction in microstate C (Ursache & Noble, 2016). The observed alterations in microstate C in lower-SES individuals may reflect a broader neural adaptation process where the brain shifts attentional allocation from internal integration towards monitoring the external environment. This adaptive change could further impair psychological functions reliant on the DMN, such as integrating self-information and cognitive goals to generate self-planning processes. Future research is needed to validate this explanation by directly testing whether these neural changes mediate the link between SES and measured cognitive deficits.

Furthermore, this study initially explored the microstate transition patterns between high and low SES groups. Specifically, compared to the high SES group, the low SES group exhibited significantly higher transition probabilities from microstate A to B and A to D, and relatively lower transition probabilities from microstate B to C and C to D. These differential patterns may suggest that socioeconomic environments shape dominant neural pathway preferences during cognitive state transitions, implying that individuals from distinct SES backgrounds develop adaptively specialized cognitive control mechanisms. For individuals in low SES, the more sufficient switching between auditory, visual, and dorsal attention networks reflected a neurocognitive adaptation characterized by a tendency to adopt bottom-up control mechanisms. In this switching process, enhanced environmental monitoring and adaptive attentional reallocation may be involved (Zhang & Lyu, 2024). According to Scarcity Theory, this switching helps low SES individuals to cope with financial stressors urgently related to current survival (Shah, Mullainathan & Shafir, 2012). The results align with evidence that heightened exposure to existential instability and environmental threats predisposes individuals with low SES to adopt bottom-up control mechanisms to focus on resolving immediate, concrete challenges (Barry et al., 2022; Zhang et al., 2025). For individuals in high SES, the more sufficient switching between the visual network, DMN, and dorsal attention network indicated a tendency to adopt top-down control mechanisms. In this switching process, focusing on internal psychological feelings helped high SES individuals enable smooth transitions between introspective and goal-directed states (Bréchet et al., 2019). Based on Construal Level Theory and Life History Theory, individuals from high SES backgrounds are less burdened by survival-related economic concerns and free up cognitive bandwidth (Griskevicius et al., 2011; Trope & Liberman, 2010). They possess greater cognitive resources to engage in future-oriented planning and abstract goal formulation, consequently favoring top-down control strategies that emphasize long-term objectives (Tomiyama et al., 2022; Zhang et al., 2025). The differential transition patterns in microstates for high and low SES groups suggested that socioeconomic environments shape divergent cognitive control mechanisms to cope with environmental demands.

In sum, the present study suggests that using resting-state EEG microstate analysis to address environmental experience as a mechanism contributing to SES-related differences in language and cognitive development. EEG microstate analysis could provide important measures that could be used in research on socioeconomic disparities in brain development.

The present study still has several limitations. First, the study only identified SES-related differences in microstate parameters, and the association between microstate parameters and behavioral outcomes relied on extrapolations from existing literature. It remains unclear whether microstates can be used as an explanatory mechanism for SES affecting cognition due to the lack of objective assessments of cognitive function and phonological processing ability in the participants. Future studies should integrate standardized behavioral metrics to empirically validate this proposed explanatory role. Second, this study examines distal factor effects on neurocognitive outcomes *via* microstates, but the significant impact of proximal factors should not be overlooked. The present study reflected the influence of life conditions on microstates through SES, which is a distal factor that remains stable over the life course and impacts neurocognitive outcomes indirectly. However, it is important to note that the potential role of more immediate proximal factors, such as stress, may have a more direct impact on brain dynamics and individual neurocognitive outcomes (Hu et al., 2021). Future studies could explore the temporal and spatial dynamic patterns of different large-scale brain networks in individuals following the proximal factors. Third, the assessment of SES in this study may not be sufficient to represent participants’ socioeconomic profiles comprehensively. The use of parental education and occupation to measure SES is a reasonable but simplified approach. It may be limited in capturing the full complexity of SES. Given that different socioeconomic factors reflect distinct aspects of lived experience (Brito & Noble, 2014; Schneider et al., 2024). Future research should be aimed at disentangling the potential primacy of any single factor. This could be achieved by incorporating a broader set of socioeconomic indicators. Including factors such as income stability, income-to-needs ratios, or neighborhood quality allows for a more nuanced and comprehensive assessment. Finally, the extreme group approach may reduce the sample size and limit the observation of smaller differences. To examine the stability of the results, additional correlation analyses were conducted when participants across the entire range of SES were included. The main findings remain consistent. The detailed results have been included in the supplementary materials. Although this suggests sufficient power to detect moderate effects, smaller gradient differences might have remained undetected, particularly for microstates B and D. Future research could explore the detection of smaller effects by using more granular groupings within the SES range.

## Conclusions

In conclusion, this study investigates the relationship between SES and microstate. Significant differences were found between high and low SES groups in the duration, occurrence, and time coverage of auditory-language network related microstate A, as well as in the occurrence and time coverage of DMN related microstate C. Furthermore, the observation of distinct sequential network activation patterns across different SES groups. The finding reveals that differences based on SES are indicative of differences in microstate parameters based on large-scale neural network function.

##  Supplemental Information

10.7717/peerj.20697/supp-1 Supplemental Information 1 Supplementary Material

## Additional Information and Declarations

### Data Availability

The following information was supplied regarding data availability: The raw data associated with this manuscript into Zenodo. The data is publicly available and can be accessed *via* the following link: https://doi.org/10.5281/zenodo.17383586.
