# Supplementary material for: The temporal dynamics of resting-state EEG microstates reflected the differences in socioeconomic status among college students
Source: PeerJ. 2026 Jan 30;14:e20697. doi: 10.7717/peerj.20697 (PMC12863152; doi:10.7717/peerj.20697)
Supplement: Supplemental Information 1 [file peerj-14-20697-s001.docx]

**Supplementary Material for**

**The Temporal Dynamics of Resting-State EEG Microstates Reflected the Differences in Socioeconomic Status Among College Students**

Qidan Ren^*^, Fangfang Long^*^, Yunlu Xie, Huiling Chen, Ying Jiang^🖂^

^*^ These authors have contributed equally to this work.

^🖂^ Corresponding Author:

Ying Jiang

Email address: [jyingpsy@126.com](mailto:jyingpsy@126.com)

**This Word file includes:**

Results

**Results**

**Microstate Topographies for the Middle SES Groups**

Four archetypal microstate topographies A-D for the middle SES groups were identified (see Fig. S1), the GEV of the four microstates was 78.31% (*SD*=4.16%) in the middle SES group.

**
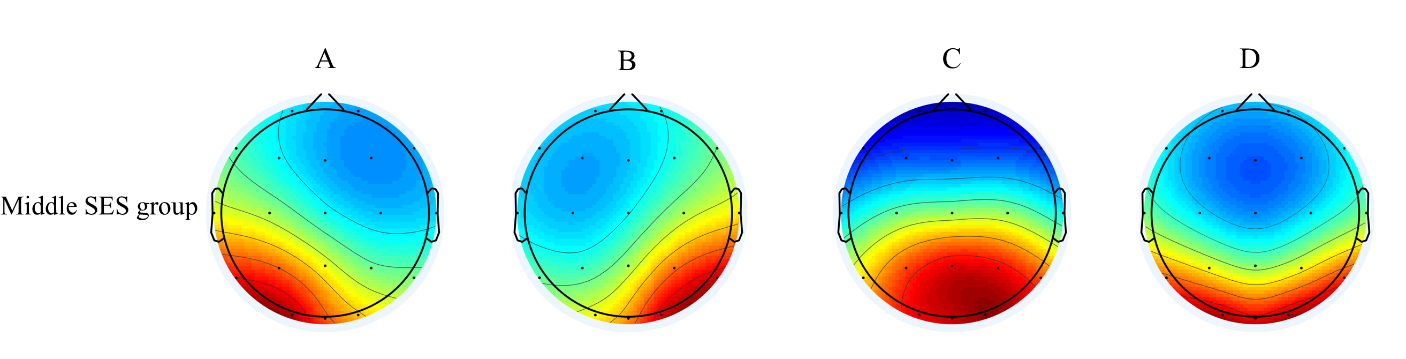
**

Fig. S1 The group average template maps of the four microstate categories in the middle SES group.

**Correlation between SES and Microstate Parameters**

Examining the association between SES and microstates across the entire range of SES. For microstate duration, no significant correlation was found between SES and the duration of the four microstates. For microstate occurrence, SES was negatively correlated with the occurrence of microstate A (*r*=-0.27，*p*=0.005) and showed a marginally significant positive association with the occurrence of microstate C (*r*=0.17，*p*=0.088). For microstate time coverage, SES was negatively correlated with the time coverage of microstate A (*r* = -0.29，*p*=0.002). No significant correlation was found between SES and the parameters of microstate B (*r=* 0.01 ~ 0.14, *p*s>0.05) or D (*r=*0.03 ~ 0.12, *p*s>0.05).
